# Markers of thrombogenesis are activated in unmedicated patients with acute psychosis: a matched case control study

**DOI:** 10.1186/1471-244X-11-2

**Published:** 2011-01-03

**Authors:** Jiří Masopust, Radovan Malý, Ctirad Andrýs, Martin Vališ, Jan Bažant, Ladislav Hosák

**Affiliations:** 1 Dept. of Psychiatry, Charles University in Prague, Faculty of Medicine in Hradec Králové, and University Hospital Hradec Králové, Czech Republic; 2 1st Dept. of Internal Medicine, Charles University in Prague, Faculty of Medicine in Hradec Králové, and University Hospital Hradec Králové, Czech Republic; 3 Institute of Clinical Immunology and Allergology, Charles University in Prague, Faculty of Medicine in Hradec Králové, and University Hospital Hradec Králové, Czech Republic; 4 Dept. of Neurology, Charles University in Prague, Faculty of Medicine in Hradec Králové, and University Hospital Hradec Králové, Czech Republic

## Abstract

**Background:**

Antipsychotic treatment has been repeatedly found to be associated with an increased risk for venous thromboembolism in schizophrenia. The extent to which the propensity for venous thromboembolism is linked to antipsychotic medication alone or psychosis itself is unclear. The objective of this study was to determine whether markers of thrombogenesis are increased in psychotic patients who have not yet been treated with antipsychotic medication.

**Methods:**

We investigated the plasma levels of markers indicating activation of coagulation (D-dimers and Factor VIII) and platelets (soluble P-selectin, sP-selectin) in an antipsychotic-naive group of fourteen men and eleven women with acute psychosis (age 29.1 ± 8.3 years, body mass index 23.6 ± 4.7), and twenty-five healthy volunteers were matched for age, gender and body mass index.

**Results:**

D-dimers (median 0.38 versus 0.19 mg/l, mean 1.12 ± 2.38 versus 0.28 ± 0.3 mg/l; P = 0.003) and sP-selectin (median 204.1 versus 112.4 ng/ml, mean 209.9 ± 124 versus 124.1 ± 32; P = 0.0005) plasma levels were significantly increased in the group of patients with acute psychosis as compared with healthy volunteers. We found a trend (median 148% versus 110%, mean 160 ± 72.5 versus 123 ± 62.5; P = 0.062) of increased plasma levels of factor VIII in psychotic patients as compared with healthy volunteers.

**Conclusions:**

The results suggest that at least a part of venous thromboembolic events in patients with acute psychosis may be induced by pathogenic mechanisms related to psychosis rather than by antipsychotic treatment. Finding an exact cause for venous thromboembolism in psychotic patients is necessary for its effective treatment and prevention.

## Background

Schizophrenia is a chronic mental disorder that affects about 1% of people worldwide. The disease tends to begin when patients are young and results in a shortened life span. Indeed, the life span among patients with schizophrenia is 20% shorter than that of the general population (61 versus 76 years, on average). Approximately 40% of mortality in schizophrenia is due to "unnatural causes", such as suicide or accidents. Somatic disorders represent the remaining 60% of "natural causes" [1].

Patients with schizophrenia have higher rates of somatic morbidity and mortality when compared with the general population [2]. This is generally explained by an unhealthy lifestyle, poor dietary habits, and the use of antipsychotic medication [3]. Physical morbidity and mortality in schizophrenia have recently been found to be increasing [4-6].

The risk for cardiovascular mortality among those with schizophrenia is increased twofold as compared with patients without schizophrenia [3]. Of the modifiable risk factors, obesity, smoking, hypertension and dyslipidemia are the most common [4].

Venous thromboembolism (VTE), which is clinically manifest as a deep vein thrombosis (DVT) or a pulmonary embolism (PE), is a multifactorial disease. The primary symptom of DVT is asymmetrical oedema of the limb, while chest pain, breathlessness, haemoptysis, syncope or tachycardia are the most important signs for pulmonary embolism [7]. The incidence of all types of thrombosis are strongly dependent on age. Among individuals up to 40 years of age, venous thromboembolism is the most common form of thrombosis. VTE is also the most common cause of morbidity and mortality in people under 40 years of age [8]. Risk factors for VTE work in the sense of the Virchow's triad: reduced blood flow, changes in the vessel wall, and changes in blood composition [9]. Most of the risk factors for thrombosis fall in the first (stasis) and third (changes in blood coagulability) group. The classification has recently changed and includes genetic and acquired VTE risk factors [8]. Cardiovascular mortality, including death from VTE, has recently become a topic of wide study among patients with schizophrenia because the safety of the treatment and the patient's quality of life are considered more important than in the past [10]. Clinically, it is important that patients treated with antipsychotic medications be treated for metabolic and cardiovascular disease and that collaborations with primary care physicians, diabetes specialists, and cardiologists be established to facilitate appropriate medical care for these patients [11,12].

Antipsychotic medication is associated with an increased risk for VTE [13]. This has been specifically shown in the low-potent first generation antipsychotics or clozapine [14-18]. There has also been increasing evidence concerning the relationship between second generation antipsychotics (olanzapine, risperidone) and VTE [19-23]. On the other hand, schizophrenia or bipolar affective disorder themselves are associated with VTE due to the increased prevalence of a sedentary lifestyle and lack of movements in this population. Obesity, sedation, hyperprolactinemia [24] or acute epinephrine secretion [25] are all factors that increase the likelihood of forming blood clots and are other important VTE risk factors seen in patients with acute psychosis. The risk of VTE is specifically increased in patients who are hospitalised or in physical restraints. The role of antipsychotic medications versus the presence of the mental disorder itself in the aetiology of VTE has not been fully clarified in patients with schizophrenia or other psychoses [26].

The primary aim of the study was to determine whether markers of thrombogenesis (D-dimers, blood factor VIII) and thrombocyte activation (sP-selectin) were activated in unmedicated patients with acute psychosis as compared with matched healthy volunteers.

## Methods

### Subjects

The patients were recruited for the study at the Dept. of Psychiatry, University Hospital in Hradec Králové. Inclusion criteria were as follows: hospitalised patients with acute psychosis (schizophrenia F20, delusional disorder F22, acute schizophreniform psychosis F23.2 according to the ICD-10 classification [27]), age between 18-55 years, unmedicated with antipsychotics, without serious medical comorbidities or a history of VTE. We excluded patients with pre-existing cardiovascular, pulmonary or neurological disease by reviewing the patients' medical records. We also conducted a comprehensive physical and laboratory check-up. This was supplemented by obtaining the patients' family history.

Healthy volunteers were recruited from the staff at the University Hospital in Hradec Králové. Healthy volunteers without any mental or serious somatic disorder were matched to the sample with respect to age, gender, weight, and body mass index (BMI). The possibility of mental illness among the volunteers was excluded by using a psychiatric examination. All participants voluntarily signed the "informed consent" form.

### Laboratory examinations

Venous blood from both the patients and healthy volunteers was taken between 7 and 9 AM after twelve hours of fasting. Laboratory examinations for markers of thrombogenesis and platelet activation are described in Table 1.

**Table 1:** Laboratory examinations.

Marker	Method
**D-dimers**	STA LIA-test^® ^D-DI (Diagnostica Stago) Normal values: <0.5 mg/l

**Factor VIII**	DG-F VIII (Grifols) APTT (C.K. PREST, Diagnostica Stago) Normal values: 50-150% of the factor activity

**sP-selectin**	ELISA method (R&D Systems) Normal values: 82 ± 31 ng/ml

APTT - activated partial thromboplastin time; ELISA - enzyme-linked immunosorbent assay; LIA - isoturbidimetric method

### Statistical analysis

We compared values of descriptive statistics (age, gender, weight, body mass index) between the patients and healthy controls using the Student's t-test. As for laboratory assessments in patients versus healthy volunteers, we used the Mann-Whitney U Test and Student's t-test.

### Ethical aspects

All aspects of the present study were approved by the Ethical Committee of the University Hospital in Hradec Králové. Even if patients signed the written "Informed Consent" in a state of acute psychosis, all also agreed to continue in the study in remission later on.

## Results

Twenty-five (women N = 11) patients with acute psychosis (schizophrenia N = 19; acute schizophreniform psychosis N = 5; delusional disorder N = 1) were included in the study. The control subjects were matched to the patients with respect to age, gender, weight, and body mass index. We did not find any significant demographic difference between the patients and healthy volunteers (P = NS; Student's t-test). Demographic data on patients and healthy volunteers are shown in Table 2.

**Table 2:** Demographic data on patients and healthy volunteers.

	Patients (N = 25; women N = 11)			Healthy volunteers (N = 25; women N = 12)			
		
	Average (SD)	Median	Range	Average (SD)	Median	Range	p-value (T-test)
**Age (years)**	29.1 (8.3)	28	18-52	29.3 (8.3)	28	19-53	NS

**Weight (kg)**	69.5 (14.7)	70	39-102	72.4 (12.7)	67	55-101	NS

**BMI**	23.6 (4.7)	23	16.2-39.8	23.5 (2.6)	23.5	19.3-29.6	NS

**Duration of** **untreated** **psychosis (months)**	12.3 (18.4)	1.3	0.25-60	-	-	-	-

BMI - Body Mass Index, NS - non-significant, SD - standard deviation. T-test - Student's t-test

Plasma levels of D-dimer and sP-selectin were significantly higher in the patients as compared with the healthy volunteers (U = 157,000; p = 0.003 and U = 133,000; p = 0.0005, respectively; Mann-Whitney U Test). The plasma level of D-dimer was pathologically increased (> 0.5 mg/l) in ten patients and in only two healthy controls. We found a trend (t = 1,911; df = 46; p = 0,062; Student's T-test) towards increased levels of factor VIII in patients with psychosis as compared with healthy volunteers. Laboratory data in patients and healthy volunteers are presented in Table 3.

**Table 3:** Laboratory assessments in patients and healthy volunteers

	Patients (N = 25)			Healthy volunteers (N = 25)			
		
	Average (SD)	Median	Range	Average (SD)	Median	Range	p-value (Test)
**D-dimers** **(mg/l)**	1.12 (2.38)	0.38	0.12-11.81	0.28 (0.3)	0.19	0.04-1.6	0.003 (MW-U)

**Factor VIII (%)**	160 (72.5)	148	73-364	123 (62.5)	110	69-290	0.062 (T-test)

**sP-selectin** **(ng/ml)**	209.9 (124)	204.1	63.3-654.4	124.1 (32)	112.4	85.6-214.3	0.0005 (MW-U)

MW-U - Mann-Whitney U Test, SD - standard deviation, T-test - Student's t-test

## Discussion

Venous thromboembolism may not always be adequately recognised in people with a severe mental disorder. Asymptomatic VTE, decreased interest in the maintenance of good health care by either patients or their physicians, a lack of collaboration between the patient and his physicians, the patient's distrust of other medical specialists who are not psychiatrists, and finally psychiatrists' limited knowledge concerning VTE diagnostics are factors that may contribute to the underdiagnosis of VTE in patients with mental illness. At the same time, the life of the patient may be at risk, especially in the case of pulmonary embolism [28].

Data in the literature concerning markers of thrombogenesis in unmedicated schizophrenic patients is limited. Iwata et al. [29] found increased levels of serum soluble L-selectin, but not sP-selectin, in 23 unmedicated patients with schizophrenia when compared with patients with major depression (N = 17; P = 0.02) or healthy subjects (N = 36; P = 0.005).

In a study by Walsh et al. [30], patients with schizophrenia (N = 19) had increased platelet expression of surface receptors alpha(IIb) beta(IIIa) as compared with healthy controls (N = 19; P < 0.0001), which may contribute to their increased risk for cardiovascular illness.

Morioka et al. [31] reported the cases of two patients (women who were 29 and 67 years of age, respectively) with psychiatric stupor who developed venous thrombosis. While dehydration, infection and decubitus ulcers are serious physical complications of psychiatric stupor, this condition also increases the risk of deep venous thrombosis.

Our findings suggest that acute psychosis may reflect a pro-coagulatory state. D-dimers are fragments of insoluble fibrin detectable in plasma after fibrin coagulum has been cleaved by plasmin. Increased plasma level of D-dimer results from pathological activation of blood clotting and occurs following fibrinolysis. Assessment of the D-dimer plasma level is important in clinical practice to exclude the diagnosis of deep vein thrombosis or pulmonary embolism, especially in the outpatient setting [32]. The specificity of D-dimers for the assessment of VTE is limited due to the fact that increased plasma levels can also be found in various kinds of inflammation, necrosis, tumours or infections. Nevertheless, we did not find any clinical or laboratory markers of infection in our sample of patients. The increased plasma level of D-dimer in acute psychosis may be a marker of fibrinolysis in the course of pathological blood clotting, when elevated epinephrine secretion stimulates the activation of thrombocytes [25,33]. Andreescu et al. [34] described D-dimer as a risk factor for deep vein thrombosis in the Leiden Thrombophilia Study. The authors studied the association of D-dimer with the risk of deep vein thrombosis in 474 patients who were more than six months out from the diagnosis of a first DVT and in 474 age- and sex-matched controls. For D-dimer levels above the 70th percentile (130.5 ng/ml), the odds ratio (OR) for DVT was 2.2 (95% CI 1.6-2.9).

The finding of elevated sP-selectin plasma levels as a marker of inflammation and increased thrombogenesis in our patients is consistent with the process described above. Thrombocytes are involved in atherogenesis if endothelial dysfunction is also present. The membrane pro-coagulatory protein sP-selectin is produced by activated thrombocytes [35]. sP-selectin induces migration and adhesion of leukocytes as well as stimulation of endothelial cells and thrombocytes. sP-selectin plays an important role as a connecting element between inflammation and thrombosis [36]. Not only have increased plasma levels of sP-selectin been found in patients with venous thromboembolism in a short time after the acute incident [37,38], but they were also present after several months [39].

The median level of factor VIII coagulatory activity showed a trend towards being higher in patients than in healthy volunteers (median 148 versus 110%; U = 216,500; P = 0.062). The difference was statistically significant in the subgroups of women (psychotic versus healthy women; median 170 versus 111%; U = 33,000; p = 0.042). Twelve patients in contrast to six healthy volunteers had factor VIII coagulatory activity that was abnormally high (above 150%). Increased blood levels of factors II, V, and VIII are associated with an increased risk of venous thromboembolism according to the current literature [40]. Individuals with factor VIII coagulatory activity above 150 IU/dl have a threefold risk of developing VTE as compared with subjects with activity <150 IU/dl; and they are six times more likely to develop this condition than people with activity <100 IU/dl. People with factor VIII coagulatory activity equal to 150 IU/dl are at a 2.7-fold increased risk of venous thromboembolism when compared with the general population [41].

The authors are aware of limitations of the results. The study sample is small, so the results may only be considered as preliminary at this moment. The generalisibility of the findings may also be limited by the disease heterogeneity in psychosis.

## Conclusions

Since the 1950s, when the first antipsychotic medications were developed, there have been reports of an increased prevalence of venous thromboembolism in patients treated with antipsychotics. In the last decade, this topic has received increased attention within the scientific literature. The diagnoses of schizophrenia or bipolar affective disorder themselves as well as hospitalisation or stress-induced increases in sympathetic activation and catecholamine blood levels are also pro-thrombogenic factors. In our sample of unmedicated patients with acute psychosis, we found an increased level of blood markers of the pathological activation of blood clotting and fibrinolysis, as well as activation of thrombocytes when compared with matched healthy volunteers. Prospective studies are needed to elucidate the biological mechanisms involved in the relationship between venous thromboembolism and antipsychotic medication versus the mental disorder itself.

## Competing interests

The authors declare that they have no competing interests.

## Authors' contributions

JM, RM and LH conceived of the study, participated in its design, collected data and drafted the manuscript. CA carried out the immunoassays and made substantial contributions to the analysis and interpretations of the data. MV helped draft the manuscript and participated in data collection. JB provided statistical analysis and made substantial contributions to the analysis and interpretation of the data. All authors read and approved the final manuscript.

## Pre-publication history

The pre-publication history for this paper can be accessed here:

http://www.biomedcentral.com/1471-244X/11/2/prepub
